# Coffee Restores Expression of lncRNAs Involved in Steatosis and Fibrosis in a Mouse Model of NAFLD

**DOI:** 10.3390/nu13092952

**Published:** 2021-08-25

**Authors:** Stefania Di Mauro, Federico Salomone, Alessandra Scamporrino, Agnese Filippello, Filomena Morisco, Maria Guido, Vincenzo Lembo, Valentina Cossiga, Rosaria Maria Pipitone, Stefania Grimaudo, Roberta Malaguarnera, Francesco Purrello, Salvatore Piro

**Affiliations:** 1 Department of Clinical and Experimental Medicine, University of Catania, 95122 Catania, Italy; 8stefaniadimauro6@gmail.com (S.D.M.); alessandraska@hotmail.com (A.S.); agnese.filippello@gmail.com (A.F.); salvatore.piro@unict.it (S.P.); 2 Division of Gastroenterology, Ospedale di Acireale, Azienda Sanitaria Provinciale di Catania, 95024 Catania, Italy; federicosalomone@rocketmail.com; 3 Department of Clinical Medicine and Surgery, University of Naples “Federico II”, 80125 Naples, Italy; filomena.morisco@unina.it (F.M.); vincenzo.lembo@unina.it (V.L.); valentina.cossiga@unina.it (V.C.); 4 Department of Medicine, University of Padua, 35121 Padua, Italy; mguido@unipd.it; 5 Department PROMISE, University of Palermo, 90128 Palermo, Italy; rosariamaria.pipitone@unina.it (R.M.P.); stefania.grimaudo@unipa.it (S.G.); 6 Faculty of Medicine and Surgery, “Kore” University of Enna, 94100 Enna, Italy; roberta.malaguarnera@unikore.it

**Keywords:** NAFLD, coffee, lncRNA, Gm16551, H19

## Abstract

Background and aim: Coffee intake exerts protective effects against non-alcoholic fatty liver disease (NAFLD), although without fully cleared mechanisms. In this study we aimed to assess whether coffee consumption may influence the expression of long non-coding RNAs (lncRNAs) in the liver. Methods: C57BL/6J mice were fed a 12-week standard diet (SD), high-fat diet (HFD) or HFD plus decaffeinated coffee solution (HFD + coffee). Expression of specific lncRNAs involved in NAFLD was analyzed by real-time PCR. For the most differentially expressed lncRNAs, the analysis was also extended to their mRNA targets. Results: Decaffeinated coffee intake reduced body weight gain, prevented NAFLD, lowered hyperglycemia and hypercholesterolemia. NAFLD was associated with lower hepatic expression of Gm16551, a lncRNA inhibiting *de novo* lipogenesis, and higher expression of H19, a lncRNA promoting fibrogenesis. Coffee intake restored Gm16551 to levels observed in lean mice and downregulated gene expression of its targets acetyl coenzyme A carboxylase 1 and stearoyl coenzyme A desaturase 1. Furthermore, coffee consumption markedly decreased hepatic expression of H19 and of its target gene collagen alpha-1(I) chain; consistently, in mice fed HFD + coffee liver expression of αSMA protein returned to levels of mice fed SD. Expression of lncRNA involved in circadian clock such as fatty liver-related lncRNA 1 (FLRL1) and fatty liver-related lncRNA 2 (FLRL2) were upregulated by HFD and were also modulated by coffee intake. Conclusion. Hepatoprotective effects of coffee may be depending on the modulation of lncRNAs involved in key pathways of NAFLD onset and progression.

## 1. Introduction

Coffee is the most consumed beverage worldwide and coffee production plays a relevant role in several countries [www.fao.org]. Coffee is consumed for its taste, flavor, and its psychoactive properties. In the last decade, it has been also shown that coffee consumption associates with beneficial effects on several health outcomes [1]. 

Epidemiological studies including prospective cohorts have shown that coffee consumption may prevent type 2 diabetes mellitus (T2DM) and may confer protection against the metabolic syndrome in general [2]. Coffee consumption is inversely associated with the degree of fibrosis in subjects with non-alcoholic fatty liver disease (NAFLD) [3,4]. Among the thousand molecules contained in coffee, pre-clinical studies have showed that the main components exerting beneficial metabolic effects are those of the polyphenolic fraction, i.e., chlorogenic acids [5], whereas the molecular mechanisms by which coffee exerts hepatoprotective effects have been only in part elucidated [6]. 

Long noncoding RNAs (lncRNAs) are transcripts longer than 200 nucleotides lacking a long protein-coding open reading frame (ORFs) [7,8]. LncRNAs are involved in a myriad of cellular processes through the regulation of gene expression at epigenetic, transcriptional, post-transcriptional, translational, and post-translational levels [9]. Furthermore, they are also involved in the regulation of protein localization and activity [10]. All these functions are probably determined by the ability of lncRNAs to bind DNA, other RNAs and proteins [11]. Several lncRNAs have been associated with metabolic homeostasis and disorders related to insulin resistance [7,12,13]. 

In recent years, the involvement of specific lncRNA in metabolic pathways relevant to NAFLD including lipid metabolism, fibrosis, clock gene regulation, apoptosis and inflammation has been reported [14].

The aim of this study was to establish whether the intake of coffee might influence the liver expression of lncRNAs in a diet-induced murine model of NAFLD. 

## 2. Materials and Methods

### 2.1. Animals and Treatments

This animal study was reviewed by the Ethics Committee of the University of Naples and approved by the Italian Minister of Scientific Research (Code 2014/0013808). Twenty-four 4-week-old male C57BL/6J mice were purchased from Harlan (San Pietro al Natisone, Italy). Animals were housed randomly in wire-bottomed cages and were maintained under controlled temperature conditions of 22 ± 1 °C, with a 12 h light–dark cycle and free access to water. After 1-week’s acclimation, the mice were divided into three groups and were randomly assigned to one of the following 12-week diets: (1) standard diet (SD) *n* = 8; (2) high-fat diet (HFD) *n* = 8; (3) HFD plus decaffeinated coffee solution *n* = 8 (HFD + coffee). A detailed composition of the diets is reported in Supplementary Table S1. 

Coffee-containing beverages were prepared by filtering on a filter paper (Whatman grade 113; Merck KGaA), a mix of boiling water and decaffeinated coffee powder (4:1, *v*/*w*) (Illy Caffè). Filtered coffee was portioned and stored at −20 °C until used. In a preliminary experiment, we found that the average daily consumption of solution (water or the coffee solution) was about 3.5 mL/mouse/d. The coffee-based beverage was prepared by diluting 1.5 mL of coffee in 100 mL of water. The dose administered coffee corresponded to six cups of espresso coffee or two cups of filtered coffee for a person weighing 70 kg [15]. Food and energy intake as well as body weight were recorded weekly. Food intake was calculated based on the amount of food remaining from a known amount administered weekly. After 12 weeks of the experimental diet, the mice were fasted overnight, anesthetized by Tribromoethanol 250 mg/kg intraperitoneally and sacrificed. Blood and liver samples were harvested, processed and snap-frozen until analyses.

### 2.2. Liver Histology 

A portion of the liver was fixed in 4% formaldehyde and embedded in paraffin. Sections (5 μm thick) were obtained and stained with haematoxylin and eosin. A pathologist (M.G.) blindly evaluated liver sections. Macrovesicular steatosis was assessed at low magnification (4×) and scored as Grade 0 (between 0 and 5%), Grade 1 (between 6 and 33%), Grade 2 (between 34 and 66%) and Grade 3 (>66%). Microvesicular steatosis was evaluated at higher magnification (20×) and expressed as percentage of affected cells. Necro-inflammatory foci were scored as present or absent.

### 2.3. Biochemical Analysis and Real Time PCR

Serum ALT, total cholesterol and glucose were measured on frozen sera using automated assays following the manufacturer’s instructions (Reflotron Plus, Roche Diagnostic). 

RNA extraction was performed from murine liver tissue (20 mg) of 23 samples: 8 SD, 8 HFD, 7 HFD + coffee (one sample was lost). Total RNA was extracted by using miRNeasy mini kit (Qiagen, Milan, Italy) according to the manufacturer’s instructions [16,17]. The quantity and quality of total RNA were measured with *NanoDrop* (Thermo Fisher Scientific, Monza, Italy). Specific qPCR primers for NAFLD-associated lncRNAs and their mRNA targets were generated through Primer Blast [18] and are reported in Supplementary Table S2. Transcript expression was analyzed through real-time PCR assays by using *Power SYBR Green RNA-to-CT 1-Step kit* (Thermo Fisher Scientific) in *QuantStudio 5 Real-time PCR System* (Thermo Fisher Scientific). Gene expression fold changes (FC) were determined by applying the 2^-ΔΔCt^ method and analyzing GAPDH as endogenous control [19,20]. 

Protein was extracted from 80 mg of liver tissue with RIPA lysis buffer and Western blot was made as previously reported [21]. All the immunoblot signals were detected using the Odyssey Fc System Infrared Scanner (LI-COR Biosciences, Lincoln, NE, USA) and densitometric analyses were performed by using Odyssey software Image Studio Lite Ver 5.2. We used the following antibodies: anti α-smooth muscle actin (Cell Signaling Technology, Danvers, MA, USA) and anti β-actin (Sigma Aldrich, St. Louis, MO, USA).

### 2.4. Statistical Analysis 

Continuous variables are presented as mean ± SD or median IQR (interquartile range), based on data distribution assessed by D’Agostino and Pearson test. Statistical significance was evaluated applying the ordinary one-way ANOVA with Tukey’s multiple comparisons test. Statistical significance was established at a two-tailed *p*-value < 0.05. GraphPad Prism 8 (GraphPad Software, Inc., San Diego, CA, USA) was employed for statistical analysis and graph-figure design. 

## 3. Results

### 3.1. Metabolic Parameters and Liver Histology 

At the beginning of the study, the three groups of mice had similar body weight (Table 1). At the end of the 12-week study period, mice of both HFD-fed groups, with or without coffee, had higher body weight compared to mice fed SD (Table 1). 

Mice fed HFD + coffee had lower body weight compared to HFD + vehicle despite similar food intake (Table 1). In agreement with body weight reduction, mice fed HFD + coffee displayed lower serum levels of total cholesterol and fasting glucose compared to mice fed HFD alone, whereas ALT were not significantly different among groups (Table 1).

**Figure 1:**
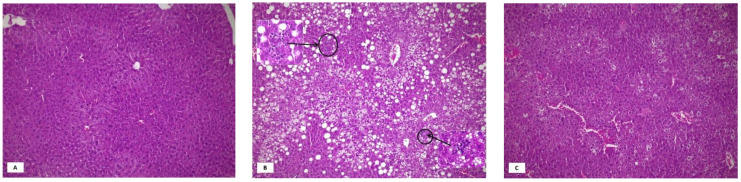
Representative pictures of liver hematoxylin-eosin staining in the three groups. Normal liver histology in mice fed standard diet (Panel (**A**), original magnification 10×). Mice on HFD for 12 weeks showed severe mixed, micro- and macrovesicular steatosis (Panel (**B**), original magnification 10×). Two necro-inflammatory foci are visible in this field (Original magnification in the inserts 40×). Amelioration of liver histology in mice fed high fat diet + decaffeinated coffee for 12 weeks: absence of macrovesicular steatosis and inflammatory foci, reduction of microvescicular steatosis (Panel (**C**), original magnification 10×).

Figure 1 shows representative pictures of liver hematoxylin-eosin staining in the three groups. All HFD animals showed some degree of steatosis, which was predominantly microvesicular in most cases (Supplementary Table S3). In coffee treated mice, macrovesicular steatosis disappeared and the degree of microvesicular steatosis was less severe, with most cases showing only Grade 1. Rare inflammatory foci were seen in four HFD mice and in none of the coffee treated animals.

### 3.2. Liver Expression of Long Non-Coding RNAs

Based on literature data, we chose 14 specific lncRNAs involved in pathways related to NAFLD onset and progression including lipid metabolism, oxidative stress, inflammation, fibrosis, circadian rhythm regulation and apoptosis. For significantly (*p* < 0.05) deregulated lncRNAs with fold change values ≤ −2 or ≥2 in HFD + coffee versus HFD, qPCR analysis was extended also to their known validated direct or indirect targets. 

### 3.3. Coffee Inhibits De Novo Lipogenesis via lncRNA Gm16551/Srebf1 Pathway

**Figure 2:**
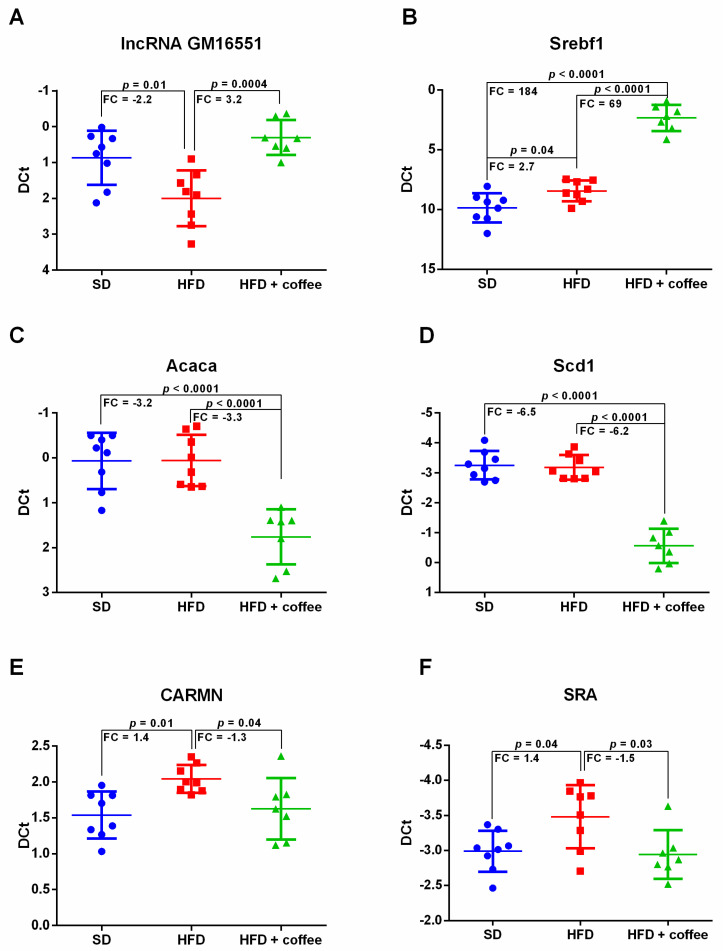
Dot plots of the hepatic expression of *Gm16551* lncRNA (Panel **A**), *sterol regulatory element-binding protein factor 1* (Srebf1) mRNA (Panel **B**), *acetyl coenzyme A carboxylase alpha* (Acaca) mRNA (Panel **C**), *stearoyl coenzyme A desaturase 1* (Scd1) mRNA (Panel **D**), *cardiac mesoderm enhancer-associated* (CARMN) lncRNA (Panel **E**) and *steroid receptor RNA activator* (SRA) lncRNA (Panel **F**), analyzed through qPCR, in mice fed standard diet (SD), high fat diet (HFD) and HFD plus decaffeinated coffee; *n* = 23: 8 SD, 8 HFD, 7 HFD + coffee. Transcript statistical significance of DE transcripts was evaluated with one-way ANOVA with Tukey post-hoc test for multiple comparisons (two-tailed *p*-value < 0.05); FC = fold change.

Figure 2 shows expression levels of Gm16551, a liver-specific lncRNA that regulates de novo lipogenesis through its interaction with the transcription factor Sterol regulatory element-binding protein isoform 1c (SREBP-1c) [22] (UniProtKB-Q9WTN3, encoded by the gene Srebf1). HFD caused a 2-fold downregulation of Gm16551 while administration of decaffeinated coffee solution determined a 3-fold upregulation of Gm16551 compared to HFD alone, restoring its expression to levels similar to SD condition (Figure 2, Panel A). 

Surprisingly, mRNA for Srebf1 displayed an increasing trend of expression from SD towards HFD to HFD + coffee conditions (Figure 2, Panel B). This may depend on the fact that we evaluated the transcript for Srebf1 instead of measuring this factor at the translational level. However, although Srebf1 mRNA was upregulated by coffee, mRNA expression of its downstream targets acetyl coenzyme A carboxylase 1 (UniProtKB-Q5SWU9 encoded by Acaca) and stearoyl coenzyme A desaturase 1 (UniProtKB-P13516 encoded by Scd1) was downregulated. In detail, the administration of coffee in HFD mice induced a 3-fold down-regulation of mRNA for Acaca in comparison both to mice fed SD and HFD + vehicle (Figure 2, Panel C). mRNA for Scd1 had a similar expression trend, with a six-fold downregulation in HFD + coffee vs. HFD alone and respect to SD (Figure 2, Panel D).

Two other lncRNAs, also involved in lipid metabolism, were slightly modified by HFD and coffee intake, the lncRNA *cardiac mesoderm enhancer-associated* (CARMN) [23] and the *steroid receptor RNA activator* (SRA) [24]. HFD induced a slight increase in the expression of CARMN and SRA versus SD, while coffee supplementation significantly decreased their expression and restored them to levels of mice fed SD (Figure 2, Panel E,F).

### 3.4. Coffee Inhibits Expression of the Fibrosis-Associated lncRNA H19

**Figure 3:**
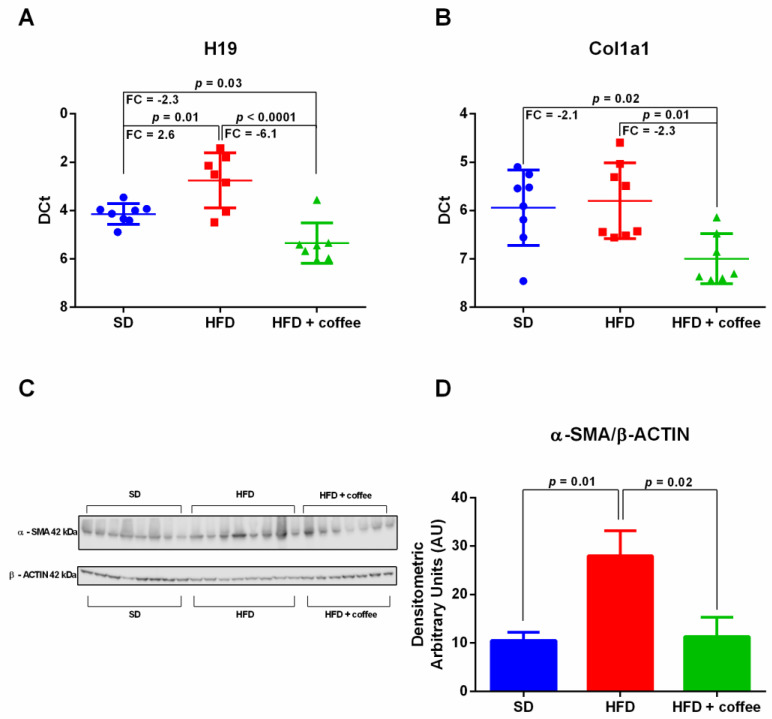
Hepatic expression of *H19* lncRNA (Panel **A**) and *Collagen alpha-1(I) chain* (Col1a1) mRNA (Panel **B**), analyzed through qPCR, in mice fed standard diet (SD), high fat diet (HFD) and HFD plus decaffeinated coffee. Transcript statistical significance was evaluated with one-way ANOVA with Tukey post-hoc test for multiple comparisons (two-tailed *p*-value < 0.05); FC = fold change. Liver expression of *alpha-smooth muscle actin* (α-SMA) protein, analyzed by Western blot (Panel **C**), and relative densitometry normalized for the housekeeping β-actin (Panel **D**). *n* = 23: 8 SD, 8 HFD, 7 HFD+ coffee.

Figure 3 shows the expression levels of *H19*, a lncRNA that is involved in liver fibrogenesis [25]. We found a 2.6 up-regulation of H19 in mice fed HFD compared to SD, whereas decaffeinated coffee reduced the expression of this lncRNA to levels lower than those observed in mice fed HFD alone and even SD (Figure 3, Panel A). We observed that mRNA for *Collagen alpha-1(I) chain* (UniProtKB-P11087 encoded by Col1a1) was downregulated in HFD + coffee in comparison to HFD and SD (Figure 3, Panel B). Although HFD is a model of early NAFLD without histological fibrosis, we also found an up-regulation of α-SMA protein expression evaluated by Western blot analysis (Figure 3, Panel C) suggesting the activation of hepatic stellate cells. In agreement with H19 down-regulation by coffee, the expression of α-SMA was restored by coffee intake to levels observed in mice fed SD (Figure 3, Panel D).

### 3.5. Coffee Modulates Expression of lncRNAs Associated with Circadian Clock Regulation

**Figure 4:**
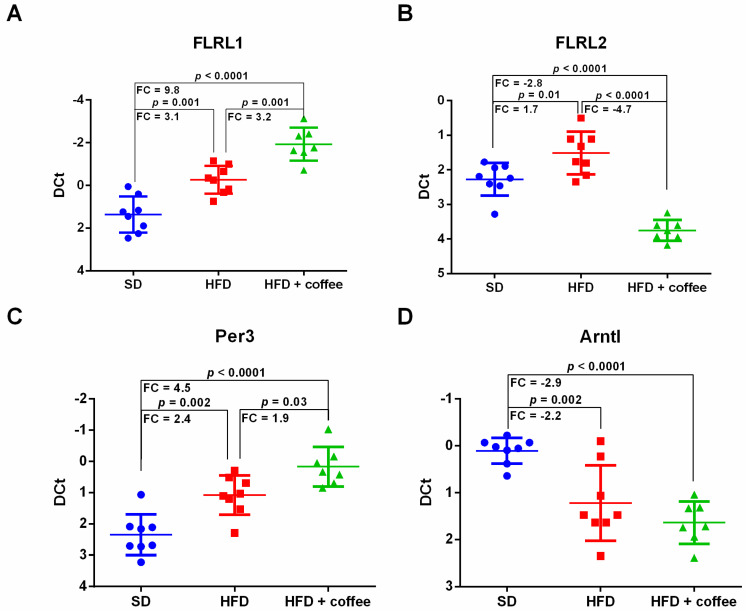
Hepatic expression of fatty liver-related lncRNA 1 (FLRL1) (Panel **A**), Fatty liver-related lncRNA 2 (FLRL2) (Panel **B**), period circadian protein homolog 3 (Per3) mRNA (Panel **C**) and Aryl hydrocarbon receptor nuclear translocator-like protein 1 (Arntl) mRNA (Panel **D**), analyzed through qPCR, in mice fed standard diet (SD), high fat diet (HFD) and HFD plus decaffeinated coffee; *n* = 23: 8 SD, 8 HFD, 7 HFD+ coffee. Transcript statistical significance was evaluated with one-way ANOVA with Tukey post-hoc test for multiple comparisons (two-tailed *p*-value < 0.05); FC = fold change.

Figure 4 shows the expression of fatty liver-related lncRNA 1 (FLRL1) and fatty liver-related lncRNA 2 (FLRL2), two lncRNAs that are involved in circadian clock regulation and whose liver expression is changed by HFD in mice [26]. Overall, we found an up-regulation of both lncRNAs in the liver of mice fed HFD versus SD, whereas their expression was differently modulated by coffee intake. In detail, coffee consumption further increased FLRL1 (Figure 4, Panel A), while FLRL2 was decreased by coffee intake to levels lower than those of mice fed SD (Figure 4, Panel B). It has been reported that FLRL1 and FLRL2 target *period circadian protein homolog 3* (UniProtKB-O70361 encoded by Per3) and *aryl hydrocarbon receptor nuclear translocator-like protein 1* (UniProtKB-Q9WTL8 encoded by Arntl), respectively [27]. Therefore, we extended qPCR analysis also to these target genes. mRNA for Per3 showed the same expression trend observed for its regulator lncRNA FLRL1; thus, it was upregulated by HFD and further increased by coffee intake (Figure 4, Panel C). Arntl mRNA expression, which was downregulated by HFD, was unaffected by coffee administration (Figure 4, Panel D).

### 3.6. lncRNAs Not Modified by Coffee Consumption

Another lncRNA involved in the regulation of metabolic processes is *colorectal neoplasia differentially expressed* (CRNDE) [28]. Although CRNDE was upregulated about three-fold by HFD, its expression was not modified by coffee consumption (Supplementary Figure S1A). Similarly, *nuclear enriched abundant transcript 1* (NEAT1), that plays a role in LDL uptake [29], was downregulated by HFD but its expression was unchanged by coffee intake (Supplementary Figure S1B). A summary of lncRNAs modified by coffee consumption and relative targets is shown in Supplementary Table S4.

## 4. Discussion

Epidemiological studies indicate that coffee intake favourably impacts on NAFLD prevalence and severity [30], although without fully clarified mechanisms. In this study we provide the first evidence that hepatoprotection induced by coffee in a mouse model is associated with the modulation of selected lncRNAs known to be involved in mechanisms related to NAFLD onset and progression such as impairment of lipid metabolism and circadian clock, pro-inflammatory state and activation of hepatic stellate cells.

Among the mechanisms connected to lipid metabolism and steatogenesis, Gm16551 has recently reported as a liver specific lncRNA downregulated in mice subjected to 24-h or a 12-week HFD that, through a negative feedback loop, reduces SREBP-1c functional activity thus inhibiting *de novo* lipogenesis [22]. In our study, Gm16551 was downregulated by a 12-week HFD, whereas coffee administration induced its expression. In agreement with histological improvement of steatosis, the induction of Gm16551 reduced the transcript for acetyl-CoA carboxylase 1 (Acaca), the enzyme that catalyzes the carboxylation of acetyl-CoA to malonyl-CoA, the first and rate-limiting step of *de novo* fatty acid biosynthesis [31]. Coffee intake reduced the mRNA level of Scd1, an enzyme that also contributes to steatogenesis [32]. Therefore, according to our data, a potential mechanism by which coffee reduces steatosis could be represented by Gm16551 expression induction. 

It is known that NAFLD is associated with a chronic inflammatory state as evidenced in the liver of animal models and patients [33]. Although the 12-week HFD is a model of early NAFLD, as showed by histology, we found a slight increase of the lncRNA CARMN that is a pro-inflammatory mediator that is upregulated in macrophages treated in vitro with high glucose and palmitic acid and in macrophages isolated from diabetic mice and whose transient overexpression stimulates the expression of inflammatory genes and of CD36 [23]. This last aspect is relevant because in HepG2 treated with palmitate, lipid overload is exacerbated by the upregulation of the receptor involved in the uptake of lipids such as CD36 [16]. Thus, in our model, the downregulation of CARMN induced by coffee administration could contribute to the observed inflammation reduction, and to the reduction of lipid uptake and consequent steatosis grade. However, the downregulation of CARMN by coffee administration could explain the complete absence of inflammatory foci in coffee treated mice. Further studies are needed to confirm this hypothesis.

Another possible contribution in this direction may rely on lncRNA SRA. In fact, it has been reported that SRA genetic knockout protects against high fat diet-induced obesity [34] and hepatic steatosis [24]. In accordance with this evidence, HFD induces the expression of lncRNA SRA, while coffee co-administration decreases its expression level with respect to HFD. Thus, SRA downregulation could contribute to the observed reduced steatosis levels. 

Since fibrosis is the main predictor of mortality in patients with NAFLD [35], it is relevant to identify molecular determinants of fibrogenesis. In this respect, experimental studies have reported the important role of the lncRNA H19. Zhu J et al. showed that H19 is overexpressed in the liver and primary hepatic stellate cells (HSCs) of mice with CCl4-induced liver fibrosis and demonstrated that the stable H19 overexpression induces the upregulation of α-SMA and Col1a1 both in vitro and in vivo [36]. Cholangiocyte-derived exosomal H19 stimulates trans-differentiation of mouse primary HSCs and induces proliferation and collagen production in HSC-derived fibroblasts [25]. In our study, we showed an up-regulation of H19 by HFD and a downregulation of H19, Col1a1 and αSMA by coffee intake. A main limitation in the interpretation of these results lies in the fact that we studied a model of early NAFLD that does not display fibrosis at H&E staining, although we cannot exclude the presence of small amount of pericellular or perisinusoidal fibrosis that could have been detected by Sirius Red. However, it is reliable to consider the upregulation of α-SMA as a marker of onset of the fibrogenesis process since in mice fed steatogenic diets the increase of α-SMA expression is confined at hepatic stellate cell level as showed by immunohistochemical analysis [37,38]. 

As concerns the circadian clock lncRNAs, Yi Chen et al., after performing a whole transcriptome analysis in an eight-week HFD mouse model, identified 266 differentially expressed lncRNA, among which they validated the expression of eight lncRNA through real time PCR [26]. To gain further insights into the molecular mechanisms regulated by such lncRNAs they performed a computational analysis that led to the identification of two fatty liver related lncRNAs associated with clock gene regulation, FLRL1 and FLRL2. They identified Per3 as a molecular target of FLRL1 computationally. The role of FLRL2 was investigated through transient inhibition in a cellular model of NAFLD; the authors demonstrated that FLRL2 downregulation is associated with Arnt downregulation at protein level [26]. However, physiological and pathophysiological functions of FLRL1 and FLRL2 and of their targets have not been elucidated so far and thus we cannot speculate on this aspect, although it deserves further exploration.

## 5. Conclusions

In this study we observed that decaffeinated coffee modulates expression of lncRNAs involved in key pathways of NAFLD onset and progression. Our data extend the knowledge concerning the molecular mechanism underlying beneficial effects exerted by coffee consumption against NAFLD.

## Figures and Tables

**Table 1:** Metabolic parameters.

Parameter (Units)	Standard Diet (SD)	High Fat Diet (HFD)	High Fat Diet (HFD) + Coffee
Initial body weight (g)	20.5 ± 1.7	20.7 ± 1.3	20.9 ± 0.7
Final body weight (g)	30.2 ± 2.5	38.4 ± 3.0 *	34.8 ± 2.5 *^†^
Food intake (Kcal/day)	4.52 ± 0.32	6.59 ± 0.38 *	6.90 ± 0.78 *
Glucose (mg/dl)	365 ± 33.6	450 ± 49.7 *	178 ± 57.6 *^†^
Total cholesterol (mg/dl)	107 ± 11.3	239 ± 42.3 *	161 ± 23.8 *^†^
ALT (IU/L)	53.3 ± 39.8	56.9 ± 16.1	45.9 ± 36.2

All variables are presented as mean ± SD because of normal distribution assessed by D’Agostino and Pearson test. Statistical significance was assessed by ordinary one-way ANOVA with Tukey’s multiple comparisons test. * *p* < 0.5 vs. SD, ^†^ *p* < 0.5 vs. HFD.

## Data Availability

Data are contained within the article or Supplementary Material.

## Supplementary Materials

The following are available online at https://www.mdpi.com/article/10.3390/nu13092952/s1, Table S1: Diet composition, Table S2: Primer sequences of selected mouse lncRNAs and relative targets, Table S3: Liver histology scores, Table S4: List of analyzed lncRNAs modulated or not modulated by coffee supplementation and relative targets, Figure S1: Dot plots of CRNDE lncRNA and NEAT1 lncRNA, analyzed through qPCR in mice fed with Standard Diet (SD), High Fat Diet (HFD) and HFD plus decaffeinate coffee *n* = 23: 8 SD, 8 HFD, 7 HFD+ coffee. Transcript statistical significance of DE transcripts was evaluated with one-way ANOVA with Tukey post-hoc test for multiple comparisons (two-tailed *p*-value < 0.05); FC= Fold Change.
