# Supplementary material for: Coffee Restores Expression of lncRNAs Involved in Steatosis and Fibrosis in a Mouse Model of NAFLD
Source: Nutrients. 2021 Aug 25;13(9):2952. doi: 10.3390/nu13092952 (PMC8467439; doi:10.3390/nu13092952)
Supplement: Supplementary file 1 [file nutrients-13-02952-s001.zip › nutrients-1273233-supplementary.pdf]

Supplementary Table S1. Diet composition.

	SD*	HFD#
Casein	210.0	265.0
L-Cystine	3.0	4.0
High Amylose Corn Starch	500.0	---
Maltodextrin	100.0	160.0
Sucrose	39.14	90.0
Anhydrous Milk fat	20.0	---
Lard	20.0	310.0
Soybean Oil	20.0	30.0
Cellulose	35.0	65.5
Mineral Mix, AIN-93G-MX (94046)	35.0	48.0
Calcium Phosphate, dibasic	---	3.4
Vitamin Mix, AIN-93-VX (94047)	15.0	21.0
Choline Bitartrate	2.75	3.0
TBHQ, antioxidant	0.01	---

Supplementary Table S2. Primer sequences of selected mouse lncRNAs and relative targets.

Gene	Forward primer	Reverse primer
GM16551	CTGAAAGCTTGTCGCGTGTC	GCGAAGGGCGAGTCGG
Srebf1	CTCAAAGACCTGGTGGTGGG	TGTGTGCACTTCGTAGGGTC
Acaca	CGATCTATCCGTCGGTGGTC	TCCTCCAGGCACTGGAACAT
Scd1	CCAAGCTGGAGTACGTCTGG	CAGAGCGCTGGTCATGTAGT
CARMN	TCATCCATCACCAAGGCCAC	CCCAAACCACCTACTCACCC
SRA	CCCACAGGATGGATCCCCTA	CACCAGTAGCAGGACAGCTC
H19	CCTCAAGATGAAAGAAATGGTGCTA	TCAGAACGAGACGGACTTAAAGAA
Col1a1	CCCCAGCCGCAAAGAGTCTA	CAGCTGACTTCAGGGATGTCT
FLRL1	CCCATCTCTGTTGGTGACCT	CGGCAAACTCAACCATTCTT
FRL2	TGGTGTGTGTGAGCCTGAGAA	AGCAGCAAGTAATAGGATGAGCAA
Per3	GCTCCGCCCCTACAGTCA	GTGTGTGCAGAGAGGACAGC
Arnlt	TCATGAGCCTCTTGGAAGCA	GGCCATGGCAAGTCACTAAAG
CRNDE	CATCAGTGTCTCGGCTCTGG	ATCACCTCCTTCCACCGGG
NEAT1	TGGAGATTGAAGGCGCAAGT	ACCACAGAAGAGGAAGCACG
MEG3	CGAGGACTTCACGCACAACA	TCCCACGCAGGATTCCAGAT
BLNC1	TGGAGATTGAAGGCGCAAGT	ACCACAGAAGAGGAAGCACG
PVT1	CCCTTTAAGCGTTCCAGAAGGA	TCGTCTGTGTGACATCTGCT
TUG1	TCTGTCCAGAACCTCAGTGC	AAGGTCATTGGCAGGTCCAG
MALAT1	TTTTGAGGGCTGACTGCCAA	GGTTGTGCTGGCTCTACCAT
LSTR	GCAAGTGGCCAGTAAATCCG	CGTTTGGGGCTCTGGATCAT
GAPDH	AGGTCGGTGTGAACGGATTTG	TGTAGACCATGTAGTTGAGGTCA

Supplementary Table S3. Liver histology scores.

Steatosis Grade	Macrovesicular Steatosis		Microvesicular Steatosis	
	HFD	HFD+Coffee	HFD	HFD+Coffee
0	2	8	3	1
1	4	0	1	5
2	1	0	0	0
3	1	0	4	2

Supplementary Table S4. List of analyzed lncRNAs modulated or not modulated by coffee supplementation and relative targets.

**LncRNAs modulated by coffee**	**Analyzed targets**
GM16551	Srebf1<br>Acaca<br>Scd1
H19	Col1a1<br>α-SMA
CARMN	/
SRA	/
FLRL1	Per3
FRL2	Arnlt
CRNDE	/
NEAT1	/
**LncRNAs not-modulated by coffee**	
MEG3	
BLNC1	
PVT1	
MALAT1	
LSTR	
GAPDH	

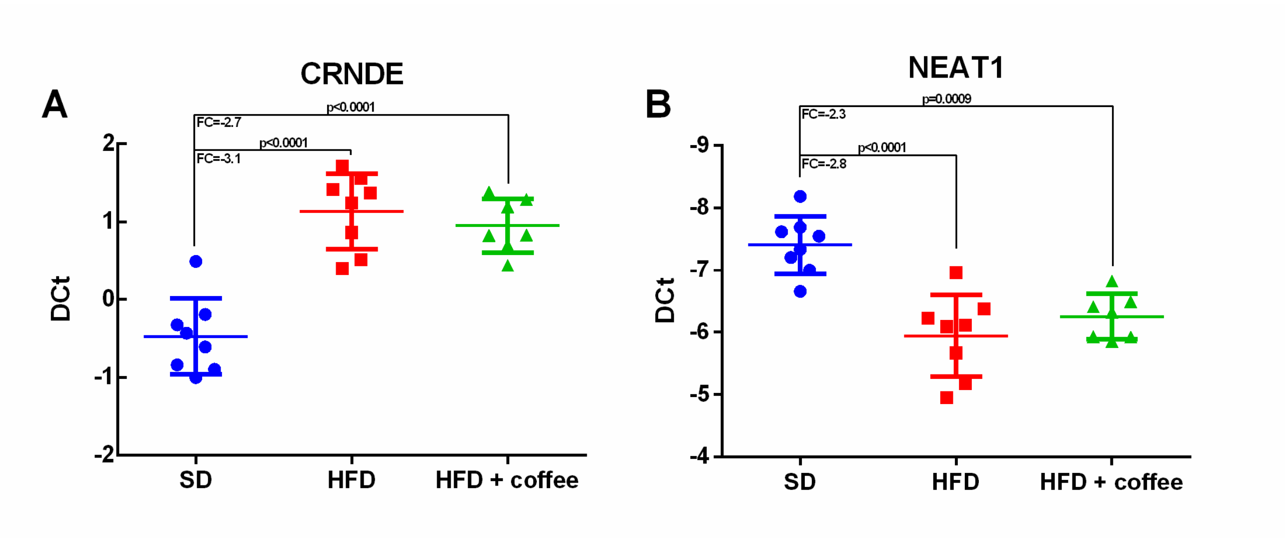

Supplementary Figure S1. Dot plots of CRNDE lncRNA and NEAT1 lncRNA, analyzed through qPCR in mice fed with Standard Diet (SD), High Fat Diet (HFD) and HFD plus decaffeinate coffee n=23: 8 SD, 8 HFD, 7 HFD+ coffee. Transcript statistical significance of DE transcripts was evaluated with one-way ANOVA with Tukey post-hoc test for multiple comparisons (two-tailed p-value < 0.05); FC= Fold Change.
